# N-acetylcysteine use in a cocaine-induced liver failure: a case report

**DOI:** 10.3389/ftox.2024.1502716

**Published:** 2025-01-15

**Authors:** Vanessa Biering, Ronan Bellouard, Maëlle Martin, Éric Dailly, Catherine Monteil-Ganière, Edouard Charles Le Carpentier

**Affiliations:** ^1^ Laboratoire de Pharmacologie Clinique, Centre Hospitalo-Universitaire (CHU) Nantes, Nantes, France; ^2^ Nantes Université, Centre Hospitalo-Universitaire (CHU) Nantes, Cibles et Médicaments des Infections et de l’Immunité, IIciMED, UR1155, Nantes, France; ^3^ Médecine Intensive Réanimation, Centre Hospitalo-Universitaire (CHU) Nantes, Nantes, France

**Keywords:** cocaine, toxicokinetics, N-acetylcysteine, intensive care unit, liver injuries

## Abstract

**Background:**

Cocaine intoxication and abuse is a worldwide problem that can be the cause of numerous acute medical complications, including severe acute hepatitis. Although these cases are scarce, they are extremely serious and may lead to liver transplantation or death. Management of toxic hepatitis, once the causative agent has been discontinued, is essentially symptomatic, based on clinical and biological monitoring and prevention of complications related to acute hepatitis.

**Case details:**

We present a case of a 28-year-old woman admitted to the emergency department for acute hepatitis due to cocaine intoxication. In addition to a sharp rise in her liver enzymes, the patient also presented metabolic acidosis, renal failure, and rhabdomyolysis. Treatment consisted of administering N-acetylcysteine (NAC), dialysis, and additional supportive measures. An improvement in the liver function with a decrease in transaminases occurred after the NAC administration. The toxicokinetics of major cocaine metabolites and clinical chemistry concentrations were monitored.

**Conclusion:**

In addition to the usual management measures for acute hepatitis, the administration of N-acetylcysteine should be investigated further, although it is currently used only in cases of acetaminophen acute toxic hepatitis.

## Introduction

Cocaine intoxication and abuse is a worldwide problem. In France, cocaine ranks second in terms of recreational illicit drug use after cannabis (Observatoire français des drogues et des tendances addictives, 2022). In addition to the cardiovascular, cerebrovascular, and kidney dysfunctions caused by cocaine consumption, few cases of hepatic dysfunction have also been reported. This hepatotoxicity typically manifests within hours to a few days after an acute overdose (Silva et al., 1991; Fernando Sierra and Diana Torres, 2004). Rarely isolated, it is usually associated with other major organ dysfunctions. The objectives of this case report are i) to describe the acute hepatic failure of a patient after cocaine intoxication, with a follow-up of cocaine and metabolite concentrations, and ii) to discuss N-acetylcysteine (NAC) use in such intoxication.

## Case details

A 28-year-old woman with a past medical history of depression and multiple addictions (tobacco, alcohol, cannabis, and cocaine) was brought to the emergency department of Nantes Hospital for tremors and disorientation following an acute cocaine intake (intake time and route unknown). The patient appeared hallucinated, with impaired alertness, bilateral mydriasis, and abnormal choreic movements (treated with diazepam and clonazepam).

The most recent psychological follow-up consultation, conducted 2 years ago, reported an addiction to cannabis and cocaine, along with a prescription history including aripiprazole, amisulpride, quetiapine, and risperidone. Upon admission, notable vital signs were a body temperature of 40°C, a heart rate of 210 beats per minute, a respiratory rate of 40 per minute, a blood pressure of 91/62 mmHg, and pulse oximetry on room air at 95%. Symptomatic management measures with external thermal control, vascular filling, and hyperhydration were carried out.

The patient was transferred to an intensive care unit (ICU) due to hemodynamic and neurological failure. The clinical symptomatology seemed compatible with a severe serotonin syndrome induced by cocaine use.

The initial biological results showed hyperlactatemia (16 mmol/L), metabolic acidosis, renal failure (creatinine = 218 μmol/L), and rhabdomyolysis (creatine kinase (CPK) = 1,518 UI/L). Troponin concentration was 1,307 ng/L.

The hemogram showed hyperleukocytosis (35 G/L), hemoglobin of 17.3 g/L, and platelets of 415 G/L. Hemostasis tests were normal. Serum acetaminophen and alcohol levels were negative.

Blood toxicology screening detected desmethyldiazepam as well as cocaine and its major metabolites, benzoylecgonine (BZE) and ecgonine methyl ester (EME). Urinary toxicological screening performed 3 h after admission revealed acetaminophen and desmethyldiazepam with an intake of cocaine and cannabis. In the emergency department, 10 mg of diazepam was administered at admission due to the patient’s agitation, and 1 g of acetaminophen was administered between 1 h and 3 h after admission for the patient’s fever. Toxicological screenings were performed with a validated method using high-performance liquid chromatography coupled with a diode array detector and single quadrupole mass spectrometry (HPLC-DAD-MS) and gas-chromatography mass spectrometry (GC-MS) after liquid–liquid extraction.

Blood quantitation of cocaine, BZE, and EME was performed with a validated method by liquid chromatography coupled with tandem mass spectrometry (LC-MS/MS) after sample deproteinization. It revealed concentrations of cocaine, BZE, and EME at 13 ng/mL, 3,760 ng/mL, and 319 ng/mL, respectively, 9 h after admission. The ethylene glycol test was negative in this sample.

After transfer to the ICU, the patient presented a multiple organ dysfunction syndrome with a severe shock requiring high doses of vasopressors. She subsequently developed hypoxia due to respiratory distress syndrome.

At 38 h post admission, biological results revealed acute hepatitis with a peak of aspartate (AST) and alanine (ALT) transaminases of 6,358 IU/L and 3,917 IU/L, respectively, associated with a severe hepatocellular insufficiency with hypoglycemia (glycemia = 3.7 mmol/L) and disseminated intravascular coagulation (prothrombin level = 22%, aPTT = 1.48, factor V = 20%, platelets = 81 G/L, and D-dimer = 44,880 ng/mL). This liver injury led to the administration of NAC at 40 h post admission. Concomitantly, metabolic acidosis persisted with hyperkalemia, increased rhabdomyolysis (CPK = 126,244 IU/L), and high troponin level (9,779 ng/L). Laboratory results and evolution related to liver and renal function are detailed in Table 1.

**TABLE 1:** Summary table of biological and toxicological panels and their evolution over 20 days.

Day	1				2			3		4		5	6	7	8	10	12	16	18	20
Post admission delay (h)	0	3	5	9	29	38	45	53	67	77	86	101	125	149	175	221	282	370	412	463
Biochemical and hemostasis results^a^																				
Creatinine (µmol/L)	218	—	235	—	338	430	442	443	371	317	392	257	410	483	683	510	801	730	—	—
AST (IU/L)	101	—	325	—	4,385	6,358	5,058	4,027	2,765	2,067	—	1,321	2,185	1,760	845	208	80	34	—	—
ALT (IU/L)	68	—	107	—	2,315	3,917	3,677	3,564	3,292	2,809	—	2,145	1,967	1,623	1,214	575	176	28	—	—
ɣ GT (UI/L)	52	—	49	—	73	71	69	68	77	75	—	93	229	276	268	194	138	108	—	—
CPK (IU/L)	1,518	—	—	—	106,720	126,244	101,500	84,052	46,517	—	—	15,305	—	—	—	2,654	—	—	—	—
PT (%)	92	—	71	—	24	22	19	17	28	—	52	70	—	115	—	115	—	—	—	—
Factor V (%)	—	—	—	—	17	20	21	32	64	—	124	>150	—	>150	—	—	—	—	—	—
Blood toxicological results^b^																				
Acetaminophen (mg/L)	<5	—	—	—	—	—	—	—	—	—	—	—	—	—	—	—	—	—	—	—
Alcohol (g/L)	<0.1	—	—	—	—	—	—	—	—	—	—	—	—	—	—	—	—	—	—	—
Ethylene glycol (mg/L)	—	—	—	<20	—	—	—	—	—	—	—	—	—	—	—	—	—	—	—	—
Cocaine (ng/mL)	—	—	—	13	—	—	ND	—	ND	ND	ND	ND	ND	ND	ND	ND	ND	—	ND	—
BZE (ng/mL)	—	—	—	3,760	—	—	2,260	—	1,161	795	942	370	305	156	143	45	18	—	3	—
EME (ng/mL)	—	—	—	319	—	—	48	—	22	17	15	10	9	5	4	ND	ND	—	ND	—
Cocaethylene (ng/mL)	—	—	—	ND	—	—	ND	—	ND	ND	ND	ND	ND	ND	ND	ND	ND	—	ND	—
Urine toxicological results^b^																				
Cocaine (ng/mL)	—	3,260	—	—	—	—	—	—	—	—	—	—	—	—	—	—	—	—	—	<1
BZE (ng/mL)	—	34,100	—	—	—	—	—	—	—	—	—	—	—	—	—	—	—	—	—	7
EME (ng/mL)	—	37,300	—	—	—	—	—	—	—	—	—	—	—	—	—	—	—	—	—	4
Cocaethylene (ng/mL)	—	ND	—	—	—	—	—	—	—	—	—	—	—	—	—	—	—	—	—	ND

Note. ND, not detectable (below detection limit); AST, aspartate transaminase; ALT, alanine transaminase; ɣ GT, gamma-glutamyltranspeptidase; CPK, creatine kinase; PT, prothrombin; BZE, benzoylecgonine; EME, ecgonine methyl ester.

^**a**^ Biochemical tests were performed using enzymatic methods (COBAS^®^ 8000 [Roche^®^ Diagnostic, Manheim, Germany]).

Hemostasis tests were performed using chronometric methods (ACL 750^®^ [Werfen^®^ analyzer, Barcelona, Spain]).

^**b**^ Blood and urine toxicological quantitation was performed using high-performance liquid chromatography coupled (HPLC LC-20AD XR [Shimatzu, Marne-la-Vallée, France]) with tandem mass spectrometry (QTRAP, 5,500 [SCIEX, Villebon-sur-Yvette, France]).

Continuous hemofiltration was initiated on the third day due to aggravation of renal failure with high CPK, followed by intermittent hemodialysis sessions from days 4 to 14.

Abdominal ultrasound revealed non-dysmorphic hepatomegaly with no signs of portal hypertension, portal thrombosis, or Budd–Chiari, and cardiac ultrasound did not suggest a cardiac cirrhosis. Infectious investigations were negative, whereas autoimmune hepatitis panels were not tested.

From the fifth day, liver function improved rapidly, with normalization of the hemostasis parameters and a slow decrease of the cytolytic peak, returning to normal 16 days after admission.

Renal function gradually improved with interruption of dialysis on day 14. The patient was discharged from the hospital 25 days after her admission with persistent renal failure (creatinine = 186 μmol/L). Four months later, renal function was fully recovered.

**FIGURE 1:**
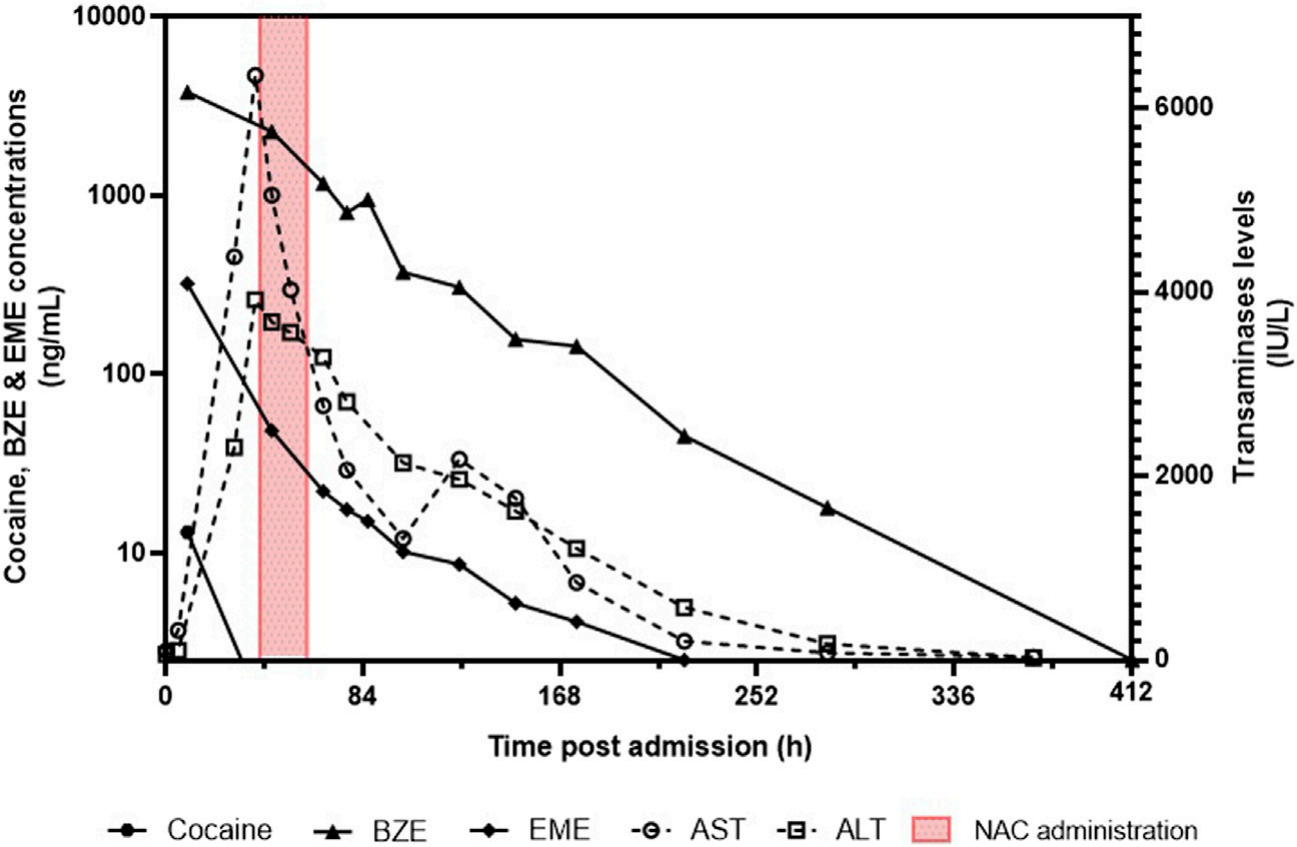
Toxicokinetics of cocaine, benzoylecgonine (BZE), ecgonine methyl ester (EME), and transaminase levels (AST and ALT).

Toxicological quantitation showed that cocaine was only detectable in plasma at 9 h after admission, whereas BZE and EME were detectable in plasma up to 18 days and 8 days after admission, respectively. Twenty days after admission, BZE and EME were still detectable in urine. The toxicokinetics of cocaine metabolites and transaminase levels are shown in Figure 1.

## Discussion

In our patient, it cannot be ruled out that liver failure was caused by a shock liver or cocaine hepatotoxicity. However, a comprehensive assessment of the patient’s symptomatology, combined with the monitoring of liver biochemical tests and the exclusion of other potential causes of liver injury, led to the diagnosis of drug-induced liver injury (DILI). Among the substances identified through toxicological analysis, some, such as acetaminophen and diazepam, had been administered during hospitalization and were unlikely to be the cause of the acute hepatitis in our case. In contrast, cocaine use is a potential cause of DILI, whereas cannabis is not known to have hepatotoxic effects (Allison et al., 2023).

At admission, quantitative determination of cocaine, BZE, and EME in blood could not be carried out because of insufficient sample volume. The first measurement of cocaine and metabolites could only be performed on a 9 h post-admission sample, showing a weak concentration of cocaine associated with high concentrations of metabolites. The presence of cocaine 9 h post admission suggests recent cocaine use in the hours before hospitalization.

Approximately 95% of cocaine is hydrolyzed to BZE (liver carboxylesterases 1) and EME (liver carboxylesterases 2 and plasma butyrylcholinesterase), both inactive metabolites (Jastrzębska and Daniel, 2023). The kinetics of BZE and EME in the blood of our patient showed a slow and regular decrease in concentrations with half-lives of 38.3 h and 26.5 h, respectively. Usually, these half-lives (intravenous route) are 7.9 h for BZE and approximately 6.8 h for EME (Ellefsen et al., 2016). This increase in the elimination half-lives was probably caused by hepatic and renal insufficiency but did not seem to be impacted by hemofiltration and dialysis.

Indeed, liver injury is attributed to the metabolic pathway mediated by cytochrome P450 3A4 (metabolizing approximately 5% of cocaine) (Jastrzębska and Daniel, 2023). The metabolites generated include N-hydroxy-norcocaine, norcocaine nitroxide, and norcocaine nitrosonium, which are not commonly measured in laboratories. These metabolites contribute to oxidative stress due to the production and the accumulation of reactive oxygen species (ROS) with a decrease in cellular antioxidants, such as glutathione (GSH), and lipid peroxidation (Jastrzębska and Daniel, 2023; Valente et al., 2012). All these processes lead to necrosis or apoptosis of liver cells.

NAC is a synthetic derivative of the non-essential amino acid cysteine, which stimulates GSH production in most cells. NAC antioxidant effects result in direct action against oxidant species, its free thiol group reacting with the reactive oxygen and nitrogen species, thereby donating electrons. NAC also has an indirect action by boosting intracellular cysteine levels, facilitating GSH synthesis (Tenório et al., 2021).

In cases of acetaminophen poisoning, NAC acts as an antidote by replenishing depleted hepatic GSH levels, which are essential for detoxification. GSH effectively neutralizes the toxic metabolite N-acetyl-p-benzoquinone imine (NAPQI) and scavenges reactive oxygen and nitrogen species (Raghu et al., 2021).

During cocaine intoxication, there is a significant increase in ROS production, leading to oxidative stress caused by the oxidation–reduction reaction of the norcocaine nitroxide/N-hydroxy-norcocaine couple. NAC may play a role in the reduction of these ROS by increasing catalase, glutathione peroxidase, and GSH synthesis (Valente et al., 2012). It has been shown in mice that pretreatment with the GSH precursor NAC exerted a protective effect against cocaine hepatotoxicity (Labib et al., 2003).

According to the French Society of Anesthesiology and Critical Care Medicine (Société Française d’Anesthesie et Réanimation, SFAR) and the French Association for the Study of the Liver (Association française pour l’étude sur le foie, AFEF) (Paugam-Burtz et al., 2020), in cases of severe acute liver failure, NAC therapy initiation is recommended to reduce morbidity and mortality regardless of the suspected etiology. These French recommendations are in accordance with the 2011 recommendations of the American Association for the Study of Liver Diseases (AASLD) (Lee et al., 2011).

These recommendations are based on studies (Hu et al., 2015) (Kortsalioudaki et al., 2008) (Squires et al., 2013) (Mumtaz et al., 2009) (Darweesh et al., 2017) (Nabi et al., 2017) (Lee et al., 2009) suggesting that NAC is associated with improved overall survival and transplant-free survival when administered during the early stages of encephalopathy in non-acetaminophen-related acute hepatitis. In these studies, NAC was administered mainly by the continuous intravenous route in varying doses.

In our case, NAC was administered 40 h after admission and with the confirmation that no acetaminophen was detected in the blood at admission. That decision was taken because of the seriousness of the liver failure and according to French guidelines on the management of liver failure in a general intensive care unit (Paugam-Burtz et al., 2020). Physicians in charge administrated NAC following the protocol recommended in France and by the AASLD for acetaminophen intoxication (a loading dose of 150 mg/kg over 15 min; a maintenance dose of 50 mg/kg given over 4 h followed by 100 mg/kg administered over 16 h) (Lee et al., 2011) (Sanabria-Cabrera et al., 2022) (Mégarbane, 2015). An improvement of the liver function with a decrease in transaminases occurred after the NAC administration and also coincided with the correction of the hemodynamic failure.

The role of NAC is firmly established in acetaminophen toxicity. Its use in intoxication to other substances that increase ROS production, like cocaine, is not as thoroughly defined and is still questioned in the medical community. This points out the need for complementary studies, including new randomized trials in cases of acute hepatitis unrelated to paracetamol, comparing NAC with placebo, with a focus on identifying predictors of response, dose, and duration of NAC.

## Conclusion

Severe acute hepatitis is a rare and potentially fatal cocaine intoxication complication and is little known in the medical community.

Our case showed the survival of the patient without sequelae after NAC administration, dialysis, and additional supportive measures. Given the mechanism of hepatotoxicity of cocaine and the relative safety of NAC, the use of this antidote should be investigated further for cocaine-induced liver failure and, more generally, for non-acetaminophen-related toxic hepatitis with oxidative stress.

## Data Availability

The raw data supporting the conclusions of this article will be made available by the authors, without undue reservation.
